# Monocular Visual/IMU/GNSS Integration System Using Deep Learning-Based Optical Flow for Intelligent Vehicle Localization

**DOI:** 10.3390/s25196050

**Published:** 2025-10-01

**Authors:** Jeongmin Kang

**Affiliations:** School of Information Technology, Halmstad University, 30118 Halmstad, Sweden; jeong.min.kang@hh.se

**Keywords:** global navigation satellite system (GNSS), Kalman filter, localization, multi-sensor fusion, optical flow, visual-inertial odometry

## Abstract

Accurate and reliable vehicle localization is essential for autonomous driving in complex outdoor environments. Traditional feature-based visual–inertial odometry (VIO) suffers from sparse features and sensitivity to illumination, limiting robustness in outdoor scenes. Deep learning-based optical flow offers dense and illumination-robust motion cues. However, existing methods rely on simple bidirectional consistency checks that yield unreliable flow in low-texture or ambiguous regions. Global navigation satellite system (GNSS) measurements can complement VIO, but often degrade in urban areas due to multipath interference. This paper proposes a multi-sensor fusion system that integrates monocular VIO with GNSS measurements to achieve robust and drift-free localization. The proposed approach employs a hybrid VIO framework that utilizes a deep learning-based optical flow network, with an enhanced consistency constraint that incorporates local structure and motion coherence to extract robust flow measurements. The extracted optical flow serves as visual measurements, which are then fused with inertial measurements to improve localization accuracy. GNSS updates further enhance global localization stability by mitigating long-term drift. The proposed method is evaluated on the publicly available KITTI dataset. Extensive experiments demonstrate its superior localization performance compared to previous similar methods. The results show that the filter-based multi-sensor fusion framework with optical flow refined by the enhanced consistency constraint ensures accurate and reliable localization in large-scale outdoor environments.

## 1. Introduction

Advancements in autonomous driving sensor performance and efficient processing capabilities for large-scale multi-sensor data have contributed significantly to the development of autonomous vehicle technology. These improvements have also facilitated the widespread adoption of deep learning-based approaches, which effectively address the limitations of traditional methods and improve robustness in complex environments. A typical autonomous driving architecture utilizing onboard sensors consists of perception, localization and mapping, path planning, decision-making, and vehicle control [1]. To achieve a fully autonomous driving system, each component must maintain accuracy and completeness while operating in a seamlessly integrated manner. In particular, localization, which estimates the position of the vehicle, is crucial for both ensuring the performance of autonomous driving systems and enabling safe navigation in urban environments [2]. However, achieving accurate and drift-free vehicle localization remains challenging due to sensor noise, signal degradation, and environmental factors. This challenge has motivated extensive research into methods capable of robustly integrating multiple sensor modalities, including visual, inertial measurement unit (IMU), and global navigation satellite system (GNSS) data, to overcome the limitations of conventional approaches.

The widely used approach for vehicle localization involves fusing in-vehicle sensor data with satellite-based information. A navigation system integrating GNSS and IMU has been extensively utilized in various vehicle localization algorithms [3,4,5]. The IMU, an onboard vehicle sensor, continuously tracks position and orientation at a high frame rate from a given initial position. However, it suffers from drift over time due to accumulated errors. In contrast, GNSS provides global positioning information using satellite signals, making it free from drift errors. However, GNSS-based navigation systems are susceptible to environmental factors such as multipath interference or signal outages. The GNSS/IMU fusion method mitigates IMU drift errors by applying GNSS measurement corrections, thus improving vehicle position accuracy. This complementary relationship enables robust short-term motion estimation with long-term position corrections. However, fusion performance is heavily dependent on the reliability of GNSS updates, which can vary significantly with the surrounding environment. In environments such as tunnels or dense urban areas with tall buildings, satellite signals remain susceptible to interference, leading to deterioration in the performance of GNSS/IMU integration [6,7]. As alternative positioning systems, pseudolite-based approaches have been investigated to complement GNSS in urban and smart city environments [8]. Nevertheless, in such challenging urban environments, GNSS/IMU integration alone is insufficient to ensure reliable localization, highlighting the need for additional sophisticated fusion strategies.

To overcome these limitations, intelligent vehicles can improve navigation accuracy by fusing sensor data from onboard sensors, with visual–inertial odometry (VIO) emerging as a promising approach for multi-sensor integration and heterogeneous data fusion. VIO, which fuses IMU data to solve the scale recovery problem in monocular camera-based visual odometry (VO), estimates the pose of the sensors by compensating for IMU drift using image measurements. This technology is crucial for vehicles and is also applicable in mobile robotics and virtual reality [9]. With advancements in computer vision, VIO has evolved from traditional image feature-based approaches to more recent methods that utilize deep neural networks [10,11]. Feature-based approaches focus on extracting robust feature points that maintain consistency across image frames, achieving real-time performance [12,13]. However, factors such as featureless images, low-light conditions, and fast movement can degrade the performance of feature-based VIO [14]. In particular, feature-based VIO is limited by sparse features, sensitivity to illumination and texture variations, and can fail in challenging visual conditions, representing an unresolved problem in the field. To address such issues, learning-based methods employ deep neural networks to directly infer motion from raw image sequences, allowing them to remain effective even under poor lighting or blurred images. Approaches leveraging deep learning have been applied either as hybrids replacing parts of traditional VIO architectures or as end-to-end pose estimation methods, offering various alternatives to traditional VIO. However, existing deep optical flow-based approaches often rely on simple bidirectional flow consistency constraints that yield unreliable flow in low-texture or ambiguous regions, a problem that remains critical in challenging scenarios. Furthermore, end-to-end approaches exhibit lower pose estimation performance compared to traditional methods.

This paper proposes a multi-sensor fusion system that integrates monocular-based VIO and GNSS measurements for vehicle localization. The proposed method adopts a hybrid approach to VIO, replacing traditional image feature extraction by leveraging a deep optical flow network. The optical flow network predicts dense optical flow from image pairs and extracts robust optical flow from the predicted flow using an enhanced consistency constraint. The extracted flow is used as visual measurements, which are then fused with IMU data in the VIO system, and a multi-sensor fusion framework is formed by incorporating GNSS measurement. To integrate the sensor data and estimate the system state, an extended Kalman filter (EKF) is employed. Finally, the performance of the proposed method is validated using the publicly available KITTI dataset [15]. The main contributions of the proposed approach are summarized as follows:

- An integrated framework combining a hybrid VIO approach employing deep networks with GNSS measurements is proposed. The enhanced consistency constraint is applied to the predicted optical flow to selectively extract high-confidence measurements, which are then incorporated into the VIO framework. The framework is applied to real sensor-based datasets, enhancing scalability through sensor fusion while maintaining general applicability.
- A filter-based multi-sensor fusion strategy is proposed to enhance the localization accuracy of the vehicle. The proposed strategy can be applied to large-scale outdoor environments and prevent the accumulation of errors over time during long-term driving.
- The proposed method is tested on real-world data and compared with leading approaches. The results demonstrate the superiority of the proposed method in terms of localization accuracy.

The remainder of this paper is organized as follows: Section 2 discusses the existing works on VO/VIO and multi-sensor fusion approaches. Section 3 introduces the proposed system in detail. Section 4 focuses on presenting the experimental setup and provides a comprehensive analysis of the results. Finally, Section 5 presents the conclusion of the paper and potential future works.

## 2. Related Work

### 2.1. Representative VO/VIO Methods

Traditional feature-based VO/VIO approaches have utilized feature detectors such as SIFT [16], SURF [17], or ORB [18] to extract feature points from images. The extracted keypoints are continuously tracked across successive camera frames using tracking algorithms such as Kanade–Lucas–Tomasi (KLT) [19]. The movement of these keypoints in image pixels can be represented by sparse optical flow [20], which can then be used for camera pose estimation. Many VO/VIO methods and simultaneous localization and mapping (SLAM) techniques have been developed based on this approach. PTAM [9], which uses the FAST corner detector [21], divides the tracking and mapping sequences, applying the keypoint idea and optimizing the map using bundle adjustment. ORB-SLAM [10] overcomes the passive initialization drawback of PTAM with a parallel-thread structure divided into tracking, local mapping, and loop closure, making it one of the most widely used methods. However, monocular camera-based VO systems face the scale recovery problem, which can be overcome by combining the system with an IMU in VIO. MSCKF [22] is a filter-based approach that merges measurements from multiple camera frames as constraints. ROVIO [23] applies a direct method using photometric error in monocular VIO and updates the state using an EKF. VINS-Mono [13] is an optimization-based approach that employs IMU pre-integration [24] and uses a sliding window to maintain robust pose estimation performance. However, these feature-based approaches can still suffer from degraded VO/VIO performance due to factors such as featureless images, low-light conditions, and fast movement, which can affect the feature point extraction process.

Recent advancements in deep learning for computer vision have highlighted the use of convolutional neural networks (CNNs) as an alternative to traditional handcrafted features by applying them to images. Deep networks for end-to-end VO/SLAM have been proposed to directly predict relative poses between image frames through supervised learning [25,26,27] or unsupervised learning [28,29,30]. VINet [31] proposed a deep network architecture for learning end-to-end VIO sequences. However, in terms of pose estimation performance, end-to-end methods are inferior compared to traditional VO/VIO approaches. As a hybrid approach that combines deep learning algorithms with geometry-based VO, DF-VO [32] estimates optical flow and depth from deep networks, recovers scale, and estimates poses by applying geometry constraints. Based on DF-VO, a dynamic object-aware VO system, Dynamic VO [33], maintains the lightweight optical flow backbone while incorporating semantic segmentation to identify and filter dynamic objects. In addition, an extended DF-VO framework, Deep Depth-Flow odometry [34], integrates a high-pricision flow network, RAFT [35] with IMU data fusion. PLD-SLAM [36] proposed a method utilizing deep learning-based segmentation and K-means clustering to filter dynamic objects. However, long-term navigation sequences and large-scale outdoor environments with non-static objects in urban settings still lead to drift errors in these methods. Moreover, employing high-precision flow networks or integrating additional deep models generally increases computational overhead.

### 2.2. Global-Aware Multi-Sensor Fusion Methods

GNSS provides globally referenced location information and can be combined with various vehicle onboard sensors to address drift issues that local estimation methods may face during long-term navigation [37]. The fusion of visual, IMU, and GNSS information has shown the potential to offer long-term localization solutions in outdoor environments [38]. Recent hybrid localization frameworks combine inertial measurements with sporadic position updates to enhance robustness and reduce drift [39]. Similarly, IC-GVINS [40] integrates GNSS and visual-inertial odometry in a tightly-coupled, INS-centric framework, achieving robust real-time pose estimation even under GNSS outages. GVINS [41] tightly integrates GNSS raw measurements with VINS, performing global pose estimation in both indoor and outdoor environments. In [42], GNSS measurements are used to enhance VINS-Mono through graph optimization. However, GNSS signal-free conditions may compromise the robustness and accuracy of these approaches. The combination of VIO and GNSS can alleviate drift and scale uncertainties, but challenges still exist in system fusion due to failures of individual systems, particularly in complex urban environments.

As global recognition fusion methods using deep learning, ref. [43] proposed a method that fuses GNSS and IMU data using a recurrent neural network (RNN). In [2], an approach was proposed that uses long-short-term memory (LSTM) to fuse GNSS and IMU data for vehicle localization. In [44], an end-to-end global positioning system modeling sequential data with LSTM was proposed. These deep learning-based methods introduce novel flexibility in modeling complex sensor dynamics, but their dependency on locally collected data and limited generalization across unseen environments remain challenges. Continued efforts are needed to enhance the robustness and adaptability of these models for deployment in diverse and dynamically changing environments.

## 3. Methodology

### 3.1. Overview of Proposed System

**Figure 1:**
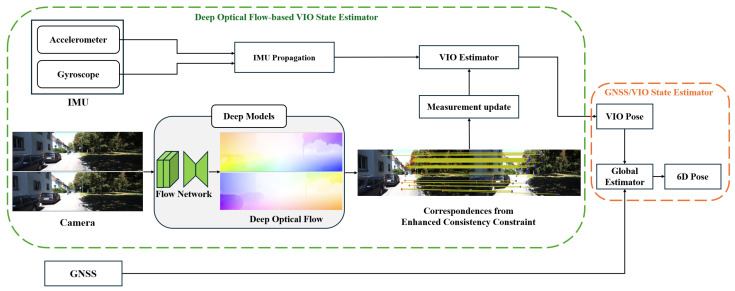
Framework of the proposed multi-sensor fusion localization system.

Figure 1 presents an overview of the proposed multi-sensor fusion system. Depending on the input sensor configuration, the system is divided into two components. The deep optical flow-based VIO state estimator fuses image and IMU data to estimate the VIO pose. IMU accelerometer and gyroscope measurements are propagated between consecutive image frames. A deep learning-based optical flow prediction network is employed to extract deep optical flow for all the image pixels. From this dense information, reliable and robust optical flow is extracted through the enhanced consistency constraint, forming the visual measurement model. The VIO estimator then fuses the IMU and camera data within the EKF framework to estimate the state of the system. The GNSS/VIO state estimator combines global GNSS data and the pose from the VIO estimator to estimate the final vehicle pose. The proposed system integrates multi-sensor information based on the EKF framework, balancing local accuracy and global consistency.

**Figure 2:**
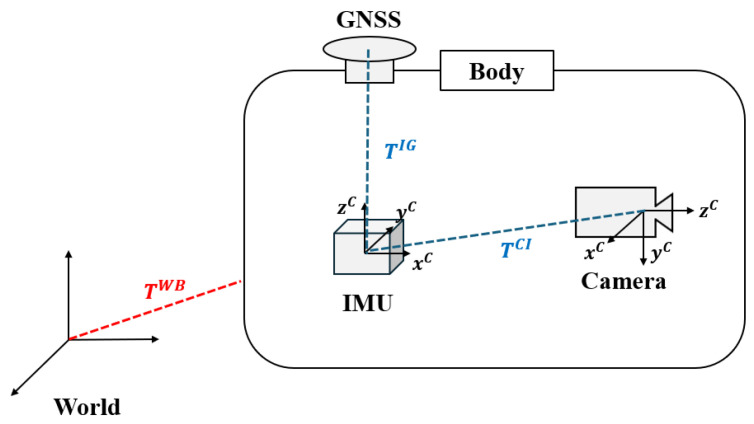
Relationships of the coordinate frames and transformation.

The IMU and camera attached to the vehicle each have their own coordinate systems, while GNSS uses earth-centered earth-fixed (ECEF) rectangular coordinates based on the Earth’s center of mass for reference. Therefore, to fuse multi-sensor data, the different coordinate systems of each sensor must be aligned, and the extrinsic parameters between the sensors are assumed to be known. The coordinate systems used in the proposed approach are defined as the world frame ${\left(\cdot \right)}^{W}$, the body frame ${\left(\cdot \right)}^{B}$, the IMU frame ${\left(\cdot \right)}^{I}$, the camera frame ${\left(\cdot \right)}^{C}$, the ECEF frame ${\left(\cdot \right)}^{E}$, and the global frame of GNSS ${\left(\cdot \right)}^{G}$. Figure 2 illustrates the relationships among the major coordinate frames. In the camera frame, the pixel frame is denoted as ${\left(\cdot \right)}^{C_{p}}$, and the normalized image frame is denoted as ${\left(\cdot \right)}^{C_{n}}$. The homogeneous representation of a point $m^{C_{p}}$ in the pixel frame is expressed as ${\tilde{m}}^{C_{p}}$. The relationship between the pixel frame and the normalized frame is defined using the intrinsic matrix from the pinhole camera model *K* as ${\tilde{m}}^{C_{n}}=K^{-1}{\tilde{m}}^{C_{p}}$ [45]. The rotation matrix $R^{\mathrm{AB}}$ represents the rotation from frame *B* to frame *A*. In this paper, it is assumed that the sensors are rigidly connected, with the camera frame defined as the body frame.

**Figure 3:**
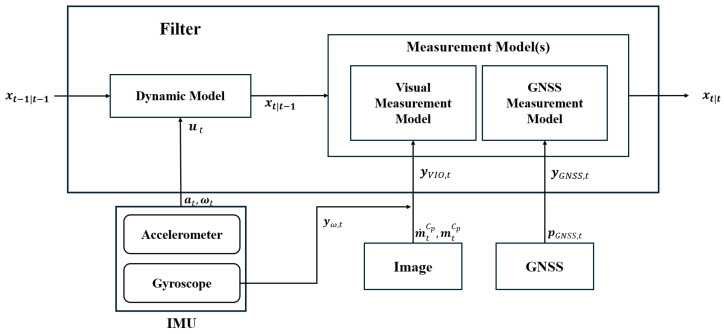
Filter architecture and data flow.

Figure 3 presents a filter architecture and the overall data flow of the proposed method. The system state is propagated using IMU measurements, including accelerometer and gyroscope data. Visual measurements are obtained from optical flow and the corresponding image pixels, combined with gyroscope data, while GNSS measurements provide global position updates. These sensor inputs are integrated within the EKF framework to produce robust and accurate vehicle pose estimates.

### 3.2. Deep Optical Flow-Based VIO

#### 3.2.1. IMU Measurement Propagation

Accounting for biased random walk and noise, the inertial measurement model for the acceleration $a_{t}^{I}$ and angular velocity $\omega_{t}^{I}$ at time *t* is formulated as:

(1) $$\begin{array}{c} \begin{array}{cc} {\hat{a}}_{t}^{I} & =a_{t}^{I}+R^{IW}g^{W}+b_{a_{t}}+n_{a},\\ {\hat{\omega }}_{t}^{I} & =\omega_{t}^{I}+b_{\omega_{t}}+n_{\omega }, \end{array} \end{array}$$

where ${\hat{a}}_{t}^{I}$ and ${\hat{\omega }}_{t}^{I}$ denote the acceleration and angular velocity measurements, respectively, and $g^{W}={\left[0,0,g\right]}^{T}$ represents the gravity vector in the world frame. Acceleration noise $n_{a}$ and angular velocity noise $n_{\omega }$ are assumed to be Gaussian white noise, given by $n_{a}∼N\left(0,\;\sigma_{a}^{2}\right)$ and $n_{\omega }∼N\left(0,\;\sigma_{\omega }^{2}\right)$, respectively. The acceleration bias $b_{a_{t}}$ and gyroscope bias $b_{\omega_{t}}$ are modeled as random walk processes, described as:

(2) $$\begin{array}{c} \begin{array}{cc} b_{a_{t}} & =b_{a_{t-1}}+n_{b_{a}},\\ b_{\omega_{t}} & =b_{\omega_{t-1}}+n_{b_{\omega }}, \end{array} \end{array}$$

where $n_{b_{a}}$ and $n_{b_{\omega }}$ represent the noise of acceleration bias and gyroscope bias, respectively, which are assumed to be Gaussian distributions, expressed as $n_{b_{a}}∼N\left(0,\;\sigma_{b_{a}}^{2}\right)$ and $n_{b_{\omega }}∼N\left(0,\;\sigma_{b_{\omega }}^{2}\right)$.

For the construction of the EKF in the proposed method, the state vector x is defined as:

(3) $$\begin{array}{c} x=\left[\begin{array}{c} p^{W}\\ v^{W}\\ q^{WB}\\ b_{a}\\ b_{\omega } \end{array}\right], \end{array}$$

where $p^{W}$ represents the position, $v^{W}$ denotes the velocity, and $q^{WB}$ is a unit quaternion that defines the orientation of the body frame *B* expressed in the world frame *W*.

Since the IMU operates at a higher frame rate than the camera, its measurements are integrated between consecutive image frames. The state propagation equations for position, velocity, and quaternion over the time interval Δt are given by:

(4) $$\begin{array}{c} \begin{array}{cc} p_{t}^{W} & =p_{t-1}^{W}+\Delta tv_{t-1}^{W}+\frac{1}{2}\Delta t^{2}\left(R_{t-1}^{WB}\left({\hat{a}}_{t}^{B}-b_{a_{t}}-n_{a}\right)-g^{W}\right),\\ v_{t}^{W} & =v_{t-1}^{W}+\Delta t\left(R_{t-1}^{WB}\left({\hat{a}}_{t}^{B}-b_{a_{t}}-n_{a}\right)-g^{W}\right),\\ q_{t}^{WB} & =\exp\left(\frac{1}{2}\Delta tS\left({\hat{\omega }}_{t}^{B}-b_{\omega_{t}}-n_{\omega }\right)\right)q_{t-1}^{WB}, \end{array} \end{array}$$

where

(5) $$\begin{array}{c} S\left(\omega \right)=\left[\begin{array}{cccc} 0 & -\omega_{x} & -\omega_{y} & -\omega_{z}\\ \omega_{x} & 0 & \omega_{z} & -\omega_{y}\\ \omega_{y} & -\omega_{z} & 0 & \omega_{x}\\ \omega_{z} & \omega_{y} & -\omega_{x} & 0 \end{array}\right]. \end{array}$$

Note that the measurements of acceleration ${\hat{a}}_{t}^{B}$ and angular velocity ${\hat{\omega }}_{t}^{B}$ are expressed in the body frame *B*, obtained by applying the rotation matrix $R^{BI}$ to transform from the IMU frame to the body frame.

The dynamic model of the state is given by:

(6) $$\begin{array}{c} x_{t}=f\left(x_{t-1},u_{t}\right)+w_{t}, \end{array}$$

where $u_{t}$ denotes a control input, and $w_{t}$ represents the process noise, with $w_{t}∼N\left(0,\;Q_{t}\right)$. Let ${\hat{x}}_{t}$ denote the estimation of state $x_{t}$ at time *t*, with $x_{t}∼N\left({\hat{x}}_{t},\;P_{t}\right)$. The time update for EKF is defined as follows:

(7) $$\begin{array}{cc} {\hat{x}}_{t|t-1} & =f\left({\hat{x}}_{t-1|t-1},u_{t}\right), \end{array}$$

(8) $$\begin{array}{cc} P_{t|t-1} & =F_{t}P_{t-1|t-1}F_{t}^{⊤}+W_{t}Q_{t}W_{t}^{⊤}, \end{array}$$

where

(9) $$\begin{array}{c} \begin{array}{cc} F_{t} & =\frac{\partial f}{\partial x}\left({\hat{x}}_{t-1|t-1},u_{t}\right), \end{array} \end{array}$$

(10) $$\begin{array}{c} \begin{array}{cc} W_{t} & =\frac{\partial f}{\partial w}\left({\hat{x}}_{t-1|t-1},u_{t}\right), \end{array} \end{array}$$

here, $P_{t}$ is the estimated error covariance matrix and $Q_{t}$ represents the noise covariance matrix of the process. $F_{t}$ is the Jacobian of the process model with respect to x, and $W_{t}$ is the Jacobian of the process model with respect to w.

#### 3.2.2. Enhanced Corresponding Optical Flow from Deep Network

Given an image pair $\left(I_{k},I_{k+1}\right)$, optical flow describes the 2D to 2D motion of a pixel in image $I_{k}$ to its corresponding location in image $I_{k+1}$. A deep learning-based optical flow network learns pixel-wise correspondences and estimates dense optical flow for the entire image. Since obtaining labeled ground truth data for all pixels is challenging, the network is trained and evaluated using synthetic image datasets. Advances in deep learning-based optical flow network models have surpassed the accuracy of traditional hand-crafted methods.

Accurately estimated optical flow enables robust state estimation through measurement updates. However, advanced flow prediction models designed for high accuracy often involve deep architectures incorporating RNN or attention mechanisms from transformer networks, which increase inference time [46]. Based on the comparative analysis of deep optical flow models and traditional approaches presented in [47], the proposed method incorporates PWC-Net [48] into the VIO framework. Following standard optical flow training conventions [49], the model is trained and fine-tuned using synthetic datasets and subsequently fine-tuned on the KITTI dataset.

However, using the optical flow of all pixels as measurements can be computationally expensive and degrade performance due to the inclusion of noise. To address this limitation, a consistency-based filtering method is often employed [32]. Given an image pair, the network predicts bidirectional forward–backward optical flow. The corresponding flows are compared to assess the reliability of the estimated motion at each pixel, which yields the flow difference. This approach assumes that correct correspondences should yield nearly inverse flows, i.e., they should cancel each other out when composed. Nevertheless, this consistency constraint applies the same criterion to all pixels without considering their motion context or underlying appearance. As a result, it can derive unreliable flows in low-texture areas and overlook spurious flows caused by motion ambiguity or occlusion areas. In addition, in regions with weak gradients or subtle motion signals, minor inconsistencies are more indicative of inherent uncertainty rather than genuine estimation errors.

To improve the reliability of visual measurements derived from dense optical flow, an enhanced flow consistency metric is proposed that incorporates both local image structure and motion coherence. In contrast to relying solely on bidirectional flow agreement, the proposed approach introduces a normalization strategy that accounts for spatial texture strength, motion magnitude, and neighborhood-level flow uniformity. This enables a more context-aware evaluation of flow reliability, leading to a normalized flow consistency score that reflects local structural and motion characteristics. The enhanced flow consistency score $D_{k}$ is defined as:

(11) $$\begin{array}{c} D_{k}\left(m_{k}^{Cp}\right)=\frac{\left|F_{k}^{k+1}\left(m_{k}^{Cp}\right)+F_{k+1}^{k}\left(m_{k}^{Cp}+F_{k}^{k+1}\left(m_{k}^{Cp}\right)\right)\right|}{\epsilon +\alpha \cdot ∥\nabla I_{k}\left(m_{k}^{Cp}\right)∥+\beta \cdot ∥F_{k}^{k+1}\left(m_{k}^{Cp}\right)∥+\gamma \cdot C_{k}\left(m_{k}^{Cp}\right)}, \end{array}$$

where $m_{k}^{Cp}$ denotes a pixel point in frame $I_{k}$, $F_{k}^{k+1}$ is the forward flow from frame $I_{k}$ to $I_{k+1}$, and $F_{k+1}^{k}$ is the backward flow. The term $m_{k}^{Cp}+F_{k}^{k+1}\left(m_{k}^{Cp}\right)$ identifies the corresponding pixel in the subsequent frame. The denominator incorporates three key components: the magnitude of the image gradient $∥\nabla I_{k}\left(m_{k}^{Cp}\right)∥$, which reflects local texture richness at pixel $m_{k}^{Cp}$; the magnitude of the predicted forward flow $∥F_{k}^{k+1}\left(m_{k}^{Cp}\right)∥$, indicating motion strength; and the contextual consistency term $C_{k}\left(m_{k}^{Cp}\right)$, which evaluates the coherence of flow vectors within a local neighborhood. The contextual term $C_{k}$ is computed as:

(12) $$\begin{array}{c} C_{k}\left(m_{k}^{Cp}\right)=\frac{1}{\left|N\right|}\sum_{n\in N(m_{k}^{Cp})}∥F_{k}^{k+1}\left(m_{k}^{Cp}\right)-F_{k}^{k+1}\left(n\right)∥, \end{array}$$

where n represents a neighboring pixel within a local window $N(m_{k}^{Cp})$ centered at $m_{k}^{Cp}$. The contextual term $C_{k}$ computes the average flow deviation between the central pixel and its neighbors, thereby penalizing flow vectors that significantly differ from their surrounding motion patterns. This encourages the selection of flow estimates that are not only directionally consistent but also spatially coherent, effectively suppressing unreliable measurements in regions. Parameters α, β, and γ are weighting factors that balance the contribution of texture strength, motion magnitude, and local flow coherence, respectively, in the normalization term. The constant ϵ is introduced to prevent division by zero and to ensure numerical stability in regions with minimal gradient or motion. These hyperparameters can be tuned to emphasize specific cues depending on the characteristics of the scene or the target application. By integrating these spatial and motion-aware cues, the proposed consistency score selectively retains flow vectors that exhibit both directional agreement and contextual stability. This leads to more robust visual observations, particularly in environments with heterogeneous motion or low-texture areas.

**Figure 4:**
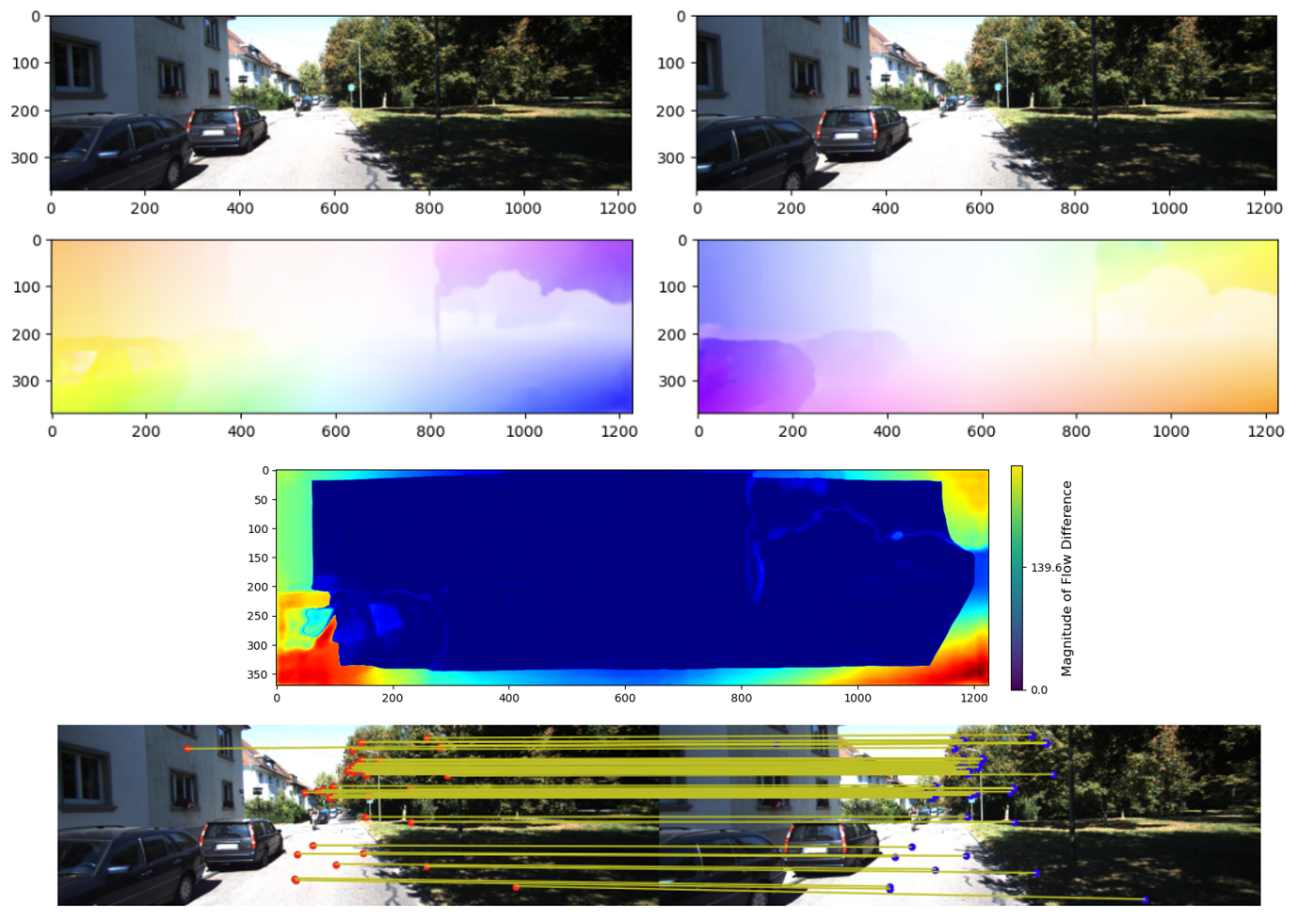
Process of extracting corresponding optical flow using a deep learning-based optical flow network.

Figure 4 illustrates the process of extracting high-confidence optical flow from two consecutive input images. The deep flow network computes dense bidirectional optical flow, and (11) is then applied to remove unreliable matches, leaving only robust flow vectors with their corresponding pixel locations. These results serve as inputs to the visual measurement model. The corresponding 2D-2D matches are illustrated in the figure to visualize the retained high-confidence optical flow.

#### 3.2.3. Visual Measurement Model

The selected optical flow in Section 3.2.2 is obtained by filtering out noisy flows from the dense optical flow of the entire image, resulting in a more accurate representation of camera motion. These optical flow measurements are then fused with the dynamic model of the IMU to estimate the state. This paper follows the derivations of [50,51] for the estimation process.

A 3D point $m^{C}$ in the camera coordinate system can be represented using the 3D point observation model that relates it to the world coordinate system, given by $m^{C}=R^{CW}m^{W}+t^{C}$. Taking the time derivative of this equation results in the following expression:

(13) $$\begin{array}{cc} {\dot{m}}^{C}\; & ={\dot{R}}^{CW}m^{W}+R^{CW}{\dot{m}}^{W}+{\dot{t}}^{C}\\ \; & ={\left[\omega^{C}\right]}_{\times }\left(m^{C}-t^{C}\right)+{\dot{t}}^{C}, \end{array}$$

where ${\dot{m}}^{C}$ denotes the optical flow in the camera frame, $R^{WC}$ and $t^{C}$ are the extrinsic parameters of the camera observation model, which transform the world coordinate system to the camera coordinate system, and ${\left[\omega^{C}\right]}_{\times }$ is the skew-symmetric matrix obtained from the angular velocity vector.

Since the measurement of the optical flow is on the image plane, the depth λ remains unknown. To solve this problem, applying the relationship $m^{C}=\lambda {\tilde{m}}^{Cn}$ to (13) leads to the following derivation:

(14) $$\begin{array}{c} {\dot{\tilde{m}}}^{Cn}=\omega^{C}\times {\tilde{m}}^{Cn}+\frac{1}{\lambda }v^{C}-\frac{\dot{\lambda }}{\lambda }{\tilde{m}}^{Cn}, \end{array}$$

where ${\dot{\tilde{m}}}^{Cn}$ is the homogeneous form of optical flow, and $v^{C}$ is related to the system states.

The depth λ can be eliminated by multiplying both sides of (14) by ${\left(v^{C}\times {\tilde{m}}^{Cn}\right)}^{T}$, resulting in:

(15) $$\begin{array}{c} 0={\left({\dot{\tilde{m}}}^{Cn}\right)}^{T}\left(v^{C}\times {\tilde{m}}^{Cn}\right)+{\left({\tilde{m}}^{Cn}\right)}^{T}\left(\omega^{C}\times \left(v^{C}\times {\tilde{m}}^{Cn}\right)\right). \end{array}$$

The measurement equation can be derived by relating the parameters to the system model. Since it is assumed that the IMU and camera are mounted on a rigid body, the angular velocity $\omega^{C}$ is obtained from the rotation matrix $R^{CI}$, which represents the transformation from the IMU frame to the camera frame. With the measurement $y_{vio,t}={\left[\begin{array}{c} {\left({\dot{\tilde{m}}}^{Cn}\right)}^{T},{\left(\omega^{C}\right)}^{T} \end{array}\right]}^{T}$ considered at time *t*, the measurement model is in the following form:

(16) $$\begin{array}{c} 0=h_{vio}\left(x_{t},y_{vio,t},e_{vio,t}\right), \end{array}$$

where $e_{vio,t}$ denotes the measurement noise, which includes the optical flow measurement noise $e^{of}$ and the angular velocity measurement noise $e^{\omega }$. Considering that the measurement noise is mutually independent at time *t*, the measurement equation is then derived as:

(17) 0=hvio(xt,yvio,t,evio,t)=(m˜˙Cn)T(vC×m˜Cn)+(m˜Cn)T(ωC×(vC×m˜Cn))=+(m˜n)T(eω×(vC×m˜Cn))+(eof)T(vC×m˜Cn).

The optical flow extracted through the enhanced consistency constraint from image pairs can have multiple values, providing additional measurement information. For each of the extracted flows, (17) is stacked, forming a comprehensive model for the measurement update. The measurement update of EKF is expressed as follows:

(18) $$\begin{array}{cc} S_{VIO,t} & =H_{VIO,t}P_{t|t-1}H_{VIO,t}^{T}+E_{t}R_{VIO,t}E_{t}^{T}, \end{array}$$

(19) $$\begin{array}{cc} K_{VIO,t} & =P_{t|t-1}H_{VIO,t}^{T}S_{VIO,t}^{-1}, \end{array}$$

(20) $$\begin{array}{cc} {\hat{x}}_{t|t} & ={\hat{x}}_{t|t-1}-K_{VIO,t}h_{VIO}\left({\hat{x}}_{t|t-1},y_{VIO,t},0\right), \end{array}$$

(21) $$\begin{array}{cc} P_{t|t} & =P_{t|t-1}-K_{VIO,t}H_{VIO,t}P_{t|t-1}, \end{array}$$

where $h_{VIO}$ is the stacked measurement model of $y_{VIO,t}$ constructed from the selected optical flow measurements in the image. $H_{VIO,t}$ represents the Jacobian of $h_{VIO}$ with respect to the state vector, $E_{t}$ represents the Jacobian of $h_{VIO}$ with respect to the noise vector $e_{vio}$, and $R_{VIO,t}$ denotes the noise covariance matrix. These matrices are defined as follows:

(22) $$\begin{array}{c} \begin{array}{cc} H_{VIO,t} & =\frac{\partial h_{VIO}}{\partial x}\left({\hat{x}}_{t|t-1},0\right), \end{array} \end{array}$$

(23) $$\begin{array}{c} \begin{array}{cc} E_{t} & =\frac{\partial h_{VIO}}{\partial e_{VIO}}\left({\hat{x}}_{t|t-1},0\right), \end{array} \end{array}$$

(24) $$\begin{array}{c} R_{VIO,t}=\left[\begin{array}{cc} \sigma_{of}^{2}I_{3} & 0_{3\times 3}\\ 0_{3\times 3} & \sigma_{\omega }^{2}I_{3} \end{array}\right]. \end{array}$$

Since the measurement includes optical flow derived from 2D camera motion on the image plane and angular velocity, the measurement update directly refines the velocity and orientation of the state. To improve position accuracy and integrate global position information, a GNSS measurement update is performed, as described in the following section.

### 3.3. GNSS Measurement Model

In general, GNSS provides absolute longitude, latitude, and altitude with respect to the Earth, making it an excellent complement to VIO. For multi-sensor fusion, the GNSS measurements provided in the global coordinate system and the VIO’s local coordinate system need to be aligned. The proposed method compares two GNSS positions measured at different times to estimate the heading angle, and integrates the gyro yaw rate over time to determine the relative heading. By comparing the gyro-based heading with the GNSS-derived heading, the initial heading can be determined, allowing the alignment of the two coordinate systems.

When the position information is received from GNSS, its measurement model is defined as follows [52]:

(25) $$\begin{array}{c} y_{p}^{G}=C_{E}^{G}p^{E}+n_{p}, \end{array}$$

where $n_{p}$ represents the Gaussian noise model for GNSS measurement noise, and $p^{E}={\left[x^{E},y^{E},z^{E}\right]}^{T}$ denotes the position in the ECEF rectangular coordinates. $p^{E}$ is obtained using the following equation:

(26) $$\begin{array}{c} \left[\begin{array}{c} x^{E}\\ y^{E}\\ z^{E} \end{array}\right]=\left[\begin{array}{c} \left(N+h_{e}\right)cos\lambda_{e}cos\phi_{e}\\ \left(N+h_{e}\right)cos\lambda_{e}cos\phi_{e}\\ {}[N(1-e^{2})+h_{e}]sin\lambda_{e} \end{array}\right], \end{array}$$

where $\left[\lambda_{e},\phi_{e},h_{e}\right]$ represent the latitude, longitude, and altitude provided by GNSS. The following parameters are defined above: $N=\frac{a}{\sqrt{1-e^{2}sin^{2}\lambda }}$ is the length from the Earth’s center to the surface; $e=\sqrt{1-\frac{b^{2}}{a^{2}}}$ is the Earth eccentricity; and the Earth’s ellipsoid semi-major and semi-minor axes are *a* = 6,378,137 m and *b* = 6,356,752.3142 m, respectively. The transition matrix $C_{E}^{G}$ from the ECEF frame to the global frame *G* is given as:

(27) $$\begin{array}{c} C_{E}^{G}=\left[\begin{array}{ccc} -sin\lambda_{e}cos\phi_{e} & -sin\lambda_{e}sin\phi_{e} & cos\lambda_{e}\\ -sin\phi_{e} & -cos\phi_{e} & 0\\ -cos\lambda_{e}cos\phi_{e} & -cos\lambda_{e}sin\phi_{e} & -sin\lambda_{e} \end{array}\right]. \end{array}$$

To fuse the GNSS measurement in the global frame with the VIO frame, the integrated system obtains the GNSS measurement model by applying $R^{WG}$ to $y_{p}^{G}$. The rotation matrix $R^{WG}$ is derived from the extrinsic parameters of the sensors. The measurement model of the GNSS is then expressed as follows:

(28) $$\begin{array}{c} y_{GNSS,t}=h_{GNSS}\left(x_{t}\right)+n_{GNSS,t} \end{array}$$

where $n_{GNSS,t}$ represents the measurement noise of the GNSS, with $n_{GNSS,t}∼N\left(0,\;R_{GNSS,t}\right)$. The measurement update is formulated as follows:

(29) $$\begin{array}{cc} S_{GNSS,t} & =H_{GNSS,t}P_{t|t-1}H_{GNSS,t}^{T}+R_{GNSS,t}, \end{array}$$

(30) $$\begin{array}{cc} K_{GNSS,t} & =P_{t|t-1}H_{GNSS,t}^{T}S_{GNSS,t}^{-1}, \end{array}$$

(31) $$\begin{array}{cc} {\hat{x}}_{t|t} & ={\hat{x}}_{t|t-1}+K_{GNSS,t}\left(y_{GNSS,t}-h_{GNSS}\left({\hat{x}}_{t|t-1}\right)\right), \end{array}$$

(32) $$\begin{array}{cc} P_{t|t} & =P_{t|t-1}-K_{GNSS,t}H_{GNSS,t}P_{t|t-1}, \end{array}$$

where $H_{GNSS,t}$ represents the Jacobian of $h_{GNSS}$ with respect to the state vector, given by:

(33) $$\begin{array}{c} \begin{array}{cc} H_{GNSS,t} & =\frac{\partial h_{GNSS}}{\partial x}\left({\hat{x}}_{t|t-1}\right). \end{array} \end{array}$$

## 4. Experimental Results

In this section, the experimental setup and results of the proposed method are described, including both quantitative and qualitative analyses.

### 4.1. Experimental Setup

The proposed method is implemented on an Intel Core i7-13620H CPU laptop (Intel Corporation, Santa lara, CA, USA) with 16 GB RAM and NVIDIA GeForce RTX 4050 laptop (NVIDIA Corporation, Santa lara, CA, USA) GPU. For the flow network, PWC-Net [48] is employed using the PyTorch 1.13.0 [53] framework, pre-trained with standard optical flow training conventions [49]. The network model is initialized with random weights and pre-trained on FlyingChairs [54]. Fine-tuning is performed on a combination of FlyingThings2D [55] and Sintel [56]. Moreover, further fine-tuning is performed on the KITTI dataset [15].

The proposed method is evaluated and compared with other methods in terms of trajectory accuracy in the KITTI dataset, which is commonly used for algorithm evaluation in autonomous driving scenarios. The sensor platform used in the dataset is equipped with two grayscale and two color cameras operating at 10 Hz, and a 100 Hz GNSS/INS navigation system for data collection. Additionally, the dataset provides ground truth camera poses. The proposed method uses a 10 Hz GNSS observation frequency. The coordinate systems of the sensors are defined as follows: the camera follows a right–down–forward convention, and the GNSS/IMU adopts a forward–left–up convention. The proposed method sets the camera as the body frame to fuse sensor data. Furthermore, most of the runtime of the proposed method was spent on optical flow network inference, with an average processing time of 156 ms per image frame.

### 4.2. Results

The proposed method is evaluated and compared with other methods in terms of localization accuracy on sequences from the KITTI odometry dataset. The methods compared in the experiments are categorized into VO, VIO, and GNSS-VIO approaches. In particular, the proposed method utilizes deep learning-based optical flow and is compared with VO methods, which are classified into pure deep learning-based methods, geometry-based methods, and hybrid methods. Pure deep learning-based methods include Depth-VO-Feat [57] and SC-SFMLearner [58], the geometry-based method is ORB-SLAM2 [12], and the hybrid methods are DF-VO [11] and Dynamic VO [33]. For VI-SLAM methods, RLD-SLAM [59] is compared, and for GNSS-VIO methods, Global-VINS [60] is compared. To further assess the impact of the enhanced correspondence, the variant that relies solely on the forward–backward flow consistency is denoted Proposed (*w*/*o* EFC), whereas the complete method including the enhanced flow consistency is denoted Proposed.

The root mean square error (RMSE) of the absolute trajectory error (ATE) is used as an evaluation metric. A smaller RMSE indicates a smaller difference between the predicted trajectory and the ground truth, making it an appropriate metric for localization evaluation. Table 1 presents the experimental results from the sequences of the KITTI dataset. In the table, ORB-SLAM2 is shown with two results, as there is a significant performance difference depending on whether loop closure is included in the algorithm. The best result for each sequence is highlighted in bold. The results indicate that the overall localization accuracy of the feature-based approach, ORB-SLAM2 with loop closure, is superior to that of pure deep learning-based VO methods. The hybrid VO approach, DF-VO, which employs optical flow and depth networks, delivers competitive performance due to scale correction from the depth network. Additionally, the multi-sensor fusion approach, integrating additional sensors, provides improved accuracy. Global-VINS, a GNSS-VIO approach, achieves higher accuracy than the VIO-based RLD-SLAM. Moreover, the results indicate that the incorporation of the enhanced flow consistency leads to improved accuracy. As shown in the RMSE results, the proposed method demonstrates strong performance compared to other methods through the multi-sensor fusion approach.

The localization results of the RMSE show that the hybrid VO approach, which incorporates a depth network, outperforms pure deep learning-based VO methods. This demonstrates the significant benefit of integrating depth information for more accurate scale estimation and trajectory estimation. Furthermore, the multi-sensor fusion approach, which combines the strengths of visual odometry with additional sensor data, shows substantial improvements in localization accuracy. The proposed method, which integrates hybrid VIO and GNSS through multi-sensor fusion, competes effectively with existing methods and demonstrates superior performance in large-scale outdoor driving environments. Notably, the proposed approach achieves robust localization without drift, even under challenging conditions, marking a significant advancement in the field of autonomous navigation.

**Figure 5:**
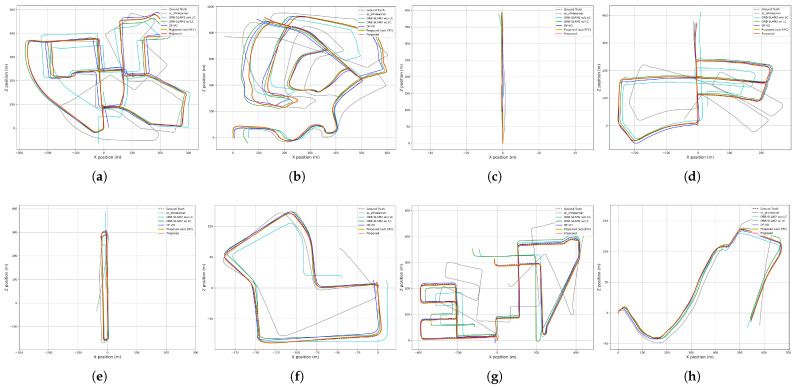
Qualitative comparison of trajectories from KITTI dataset: (**a**) Sequence 00. (**b**) Sequence 02. (**c**) Sequence 04. (**d**) Sequence 05. (**e**) Sequence 06. (**f**) Sequence 07. (**g**) Sequence 08. (**h**) Sequence 10.

To further evaluate the accuracy of pose estimation, the relative pose error (RPE) is compared. In contrast to ATE, which depends on the total number of frames in a trajectory, RPE is independent of this accumulation and evaluates frame-to-frame relative pose error. The proposed method, employing a hybrid approach with an optical flow network, computes the pose error between image frames when visual measurements are updated, allowing comparison with other visual-based approaches. Figure 5 compares the trajectories of eight sequences of the KITTI dataset, showing that the proposed method closely follows the ground truth. Table 2 presents the numerical evaluation results for RPE in terms of translation and rotation, with the best results highlighted in bold. As shown in the table, the proposed method achieves comparable or superior RPE performance compared to other methods, demonstrating strong frame-to-frame translation and rotation pose estimation accuracy.

To evaluate the global localization accuracy of the proposed method, a comparison with the GNSS data is performed. The RMSE, mean, standard deviation (Std), maximum (Max), and minimum (Min) values for the GNSS, proposed method, and ground truth, as measured in sequence 04 of the KITTI dataset, are compared and presented in Table 3. The results show that the proposed method improves the localization accuracy of the raw GNSS measurements by leveraging multi-sensor fusion. Although GNSS provides global location information without error accumulation, but still includes localization errors. By integrating additional sensor data, overall accuracy is improved. These results highlight the effectiveness of the proposed method in enhancing GNSS-based localization accuracy.

The enhanced flow consistency constraint introduced in Section 3.2.2 improves the robustness of visual measurements, as demonstrated by the accuracy gains of the proposed method over its variant without this constraint. Furthermore, the GNSS measurement model in Section 3.3 helps mitigate long-term drift in the evaluated KITTI sequences, resulting in lower RMSE values. Notably, the proposed method achieves competitive or superior performance even compared to existing GNSS-VIO approaches, underscoring the effectiveness of the fusion design. These results highlight how each design step in the filter architecture contributes to the overall localization accuracy.

The methods introduced in Section 3 are directly reflected in the results presented here. Specifically, the IMU propagation and visual measurement update (Section 3.2.1–Section 3.2.3) form the VIO estimator, while the GNSS measurement model in Section 3.3 is further integrated to constitute the complete fusion system. The enhanced flow consistency constraint introduced in Section 3.2.2 improves the robustness of visual measurements, which is validated by the accuracy gains of the proposed method over its variant without this constraint. By jointly leveraging these components, the system achieves robust state estimation, including both position and orientation through quaternion representation, leading to improved RMSE and RPE performance. Furthermore, the GNSS integration effectively mitigates long-term drift in the KITTI sequences, resulting in lower RMSE values. Notably, the proposed method achieves competitive or superior performance even compared to existing GNSS-VIO approaches, underscoring the effectiveness of the fusion design. These results highlight how each design step in the filter architecture contributes to the localization accuracy.

Furthermore, under the enhanced flow consistency constraint, more robust optical flow can be extracted from the deep prediction model. While this improves the accuracy of visual measurements and subsequent state estimation, it introduces an additional computational burden, representing a trade-off between runtime efficiency and flow prediction quality. This general consideration highlights the balance between accuracy and computational cost inherent in the use of advanced optical flow models within the proposed VIO and multi-sensor fusion framework.

Overall, the experimental results demonstrate that the proposed multi-sensor fusion method, leveraging the deep learning-based optical flow network, effectively improves vehicle localization accuracy in outdoor environments. The filter-based multi-sensor fusion strategy, which combines the hybrid VIO approach incorporating the deep optical flow network with GNSS measurements, addresses long-term error accumulation in large-scale outdoor environments by enhancing the scalability and robustness of the pose estimation framework. Tested on real-world data, the proposed method shows improvements in localization accuracy compared to standards approaches in frame-to-frame accuracy and GNSS-based localization accuracy.

## 5. Discussion and Conclusions

This paper proposes a multi-sensor fusion method that integrates monocular VIO with GNSS measurements to enhance vehicle localization accuracy in outdoor environments. Leveraging a deep learning-based optical flow network, the proposed method effectively addresses challenges related to long-term drift and error accumulation in large-scale outdoor environments. The fusion of GNSS and hybrid VIO, which employs deep optical flow, provides reliable pose estimation. The experimental evaluation on the KITTI dataset demonstrates that the proposed method outperforms similar approaches, providing strong frame-to-frame accuracy and improved GNSS-based localization performance. The results also highlight the filter-based fusion strategy, which successfully integrates data from multiple sensors to achieve an accurate and drift-free localization system.

Although the proposed method demonstrates promising results in outdoor environments, future research could extend the evaluation to more challenging real-world scenarios, such as urban canyons or tunnels, where GNSS signals are frequently degraded or completely blocked. In these environments, maintaining consistent VIO estimation and incorporating sporadic GNSS updates through tight fusion strategies could provide more robust localization performance. Such evaluations would further validate the generalization capability of the proposed framework and highlight its practical applicability under extreme navigation scenarios. In addition, the proposed method exhibits a computational bottleneck primarily in the flow inference step, where both forward and backward flows are estimated from the input images, resulting in substantial runtime overhead. To address this limitation, future work could involve strategies for reducing computational cost, including input image resampling to lower resolution prior to inference, adoption of lightweight network architectures, and selective flow estimation within regions of interest guided by IMU dynamic estimation, thereby improving the overall efficiency of the algorithm. These lightweight strategies will also be explored to further improve computational efficiency for real-time embedded systems deployment. Finally, although the evaluation of the proposed method was performed on a high-performance platform, the use of these efficiency-oriented strategies would allow deployment on typical autonomous vehicle computing units, enabling practical performance assessment in real-world navigation scenarios.

## Figures and Tables

**Table 1:** Quantitative comparison of the results on KITTI dataset (RMSE [m]). The best result is in bold.

Method	Category	00	01	02	03	04	05	06	07	08	09	10
Depth-VO-Feat [57]	Deep Learning-based VO	64.45	203.44	85.13	21.34	3.12	22.15	14.31	15.35	29.53	52.12	24.70
SC-SFMLearner [58]	Deep Learning-based VO	93.04	85.90	70.37	10.21	2.98	40.56	12.56	21.01	56.15	15.02	20.19
ORB-SLAM2 (*w*/*o* LC) [12]	Geometry-based V-SLAM	40.65	502.20	47.82	0.94	1.30	29.95	40.82	16.04	43.09	38.77	5.42
ORB-SLAM2 (w LC) [12]	Geometry-based V-SLAM	6.03	508.34	14.76	1.02	1.57	4.04	11.16	2.19	38.85	8.39	6.63
DF-VO [11]	Hybrid VO	12.17	342.71	17.59	1.96	0.70	4.94	3.73	**1.06**	6.96	7.59	4.21
RLD-SLAM [59]	VI-SLAM	1.16	0.90	0.70	**0.46**	1.28	1.50	0.17	2.34	1.01	1.49	0.74
GNSS-VINS [60]	GNSS-VIO	0.97	0.86	0.58	0.82	1.12	1.20	0.17	2.29	1.11	-	1.66
Proposed (*w*/*o* EFC)	GNSS-VIO	0.76	0.63	0.58	0.62	0.66	0.89	0.11	1.50	0.78	0.48	0.58
Proposed	GNSS-VIO	**0.65**	**0.54**	**0.50**	0.55	**0.53**	**0.80**	**0.10**	1.31	**0.67**	**0.41**	**0.51**

**Table 2:** Comparison of RPE (translation and rotation) results across sequences 00-10 of KITTI dataset.

	00	01	02	03	04	05	06	07	08	09	10
	RPE RPE	RPE RPE	RPE RPE	RPE RPE	RPE RPE	RPE RPE	RPE RPE	RPE RPE	RPE RPE	RPE RPE	RPE RPE
	(m) (°)	(m) (°)	(m) (°)	(m) (°)	(m) (°)	(m) (°)	(m) (°)	(m) (°)	(m) (°)	(m) (°)	(m) (°)
SC-SFMLearner [58]	0.14 0.13	0.89 0.08	0.09 0.09	0.06 0.07	0.07 0.06	0.07 0.07	0.07 0.07	0.08 0.07	0.09 0.07	0.10 0.10	0.11 0.11
ORB-SLAM2 (*w*/*o* LC) [12]	0.17 0.08	2.97 0.10	0.17 0.07	0.03 0.06	0.08 0.08	0.14 0.06	0.24 0.06	0.11 0.05	0.19 0.06	0.13 0.06	0.05 0.07
ORB-SLAM2 (w LC) [12]	0.21 0.09	3.04 0.09	0.22 0.08	0.04 0.06	0.08 0.08	0.29 0.06	0.73 0.05	0.51 0.05	0.16 0.07	0.34 0.06	0.05 0.07
DF-VO [11]	0.04 0.06	1.55 **0.05**	0.06 0.05	0.03 **0.04**	0.05 **0.03**	**0.02** 0.04	0.03 0.03	0.02 **0.03**	0.04 **0.04**	0.05 **0.04**	0.04 0.04
Dynamic VO [33]	**0.03** 0.06	1.71 0.67	0.04 0.06	**0.02 0.04**	0.04 0.04	**0.02** 0.05	0.03 0.05	0.02 0.04	**0.03** 0.05	0.06 0.05	0.05 0.06
Proposed (*w*/*o* EFC)	**0.03** 0.05	0.86 **0.05**	0.04 0.04	0.03 **0.04**	**0.02 0.03**	**0.02** 0.04	**0.01** 0.03	0.02 **0.03**	0.04 **0.04**	0.04 **0.04**	0.03 **0.03**
Proposed	**0.03 0.04**	**0.75 0.05**	**0.03 0.03**	0.03 **0.04**	**0.02 0.03**	**0.02 0.03**	**0.01 0.02**	**0.01 0.03**	**0.03 0.04**	**0.03 0.04**	**0.02 0.03**

**Table 3:** Comparison of GNSS and the proposed method accuracy on sequence 04 of KITTI dataset [m].

Method	RMSE	Mean	Std	Max	Min
**GNSS**	3.2766	2.9888	1.3429	5.6606	0.0164
**Proposed **	0.5703	0.5019	0.4846	3.6810	0.0059

## Data Availability

Dataset available on request from the authors.

## Abbreviations

The following abbreviations are used in this manuscript:

GNSS	Global navigation satellite system
VIO	Visual-inertial odometry
IMU	Inertial measurement units
VO	Visual odometry
EKF	Extended Kalman filter
SLAM	Simultaneous localization and mapping
CNNs	Convolutional neural networks
RNN	Recurrent neural network
LSTM	Long-short-term memory
ECEF	Earth-centered earth-fixed
RMSE	Root mean square error
ATE	Absolute trajectory error
RPE	Relative pose error
